# Ferroelectricity in Si-Doped Hafnia: Probing Challenges in Absence of Screening Charges

**DOI:** 10.3390/nano10081576

**Published:** 2020-08-11

**Authors:** Umberto Celano, Andres Gomez, Paola Piedimonte, Sabine Neumayer, Liam Collins, Mihaela Popovici, Karine Florent, Sean R. C. McMitchell, Paola Favia, Chris Drijbooms, Hugo Bender, Kristof Paredis, Luca Di Piazza, Stephen Jesse, Jan Van Houdt, Paul van der Heide

**Affiliations:** 1 Imec, Kapeldreef 75, B-3001 Heverlee (Leuven), Belgium; paolapiedimonte@gmail.com (P.P.); Mihaela.Ioana.Popovici@imec.be (M.P.); kaflorent@micron.com (K.F.); Sean.McMitchell@imec.be (S.R.C.M.); paola.favia@imec.be (P.F.); Christel.Drijbooms@imec.be (C.D.); hugo.bender@imec.be (H.B.); kristof.paredis@imec.be (K.P.); Luca.DiPiazza@imec.be (L.D.P.); Jan.VanHoudt@imec.be (J.V.H.); Paul.vanderHeide@imec.be (P.v.d.H.); 2 Institut de Ciència de Materials de Barcelona (ICMAB-CSIC), Campus UAB, Bellaterra, 08193 Catalonia, Spain; agomez@icmab.es; 3 Center for Nanophase Materials Sciences, Oak Ridge National Laboratory, 1 Bethel Valley Rd., Oak Ridge, TN 37830, USA; neumayersm@ornl.gov (S.N.); collinslf@ornl.gov (L.C.); sjesse@ornl.gov (S.J.); 4 Department of Electrical Engineering (ESAT), KU Leuven, Kasteelpark Arenberg 10, 3001 Leuven, Belgium

**Keywords:** HfO_2_-based ferroelectrics, Si-doped HfO_2_, binary oxide ferroelectrics, atomic force microscopy, band-excitation piezoresponse force microscopy

## Abstract

The ability to develop ferroelectric materials using binary oxides is critical to enable novel low-power, high-density non-volatile memory and fast switching logic. The discovery of ferroelectricity in hafnia-based thin films, has focused the hopes of the community on this class of materials to overcome the existing problems of perovskite-based integrated ferroelectrics. However, both the control of ferroelectricity in doped-HfO_2_ and the direct characterization at the nanoscale of ferroelectric phenomena, are increasingly difficult to achieve. The main limitations are imposed by the inherent intertwining of ferroelectric and dielectric properties, the role of strain, interfaces and electric field-mediated phase, and polarization changes. In this work, using Si-doped HfO_2_ as a material system, we performed a correlative study with four scanning probe techniques for the local sensing of intrinsic ferroelectricity on the oxide surface. Putting each technique in perspective, we demonstrated that different origins of spatially resolved contrast can be obtained, thus highlighting possible crosstalk not originated by a genuine ferroelectric response. By leveraging the strength of each method, we showed how intrinsic processes in ultrathin dielectrics, i.e., electronic leakage, existence and generation of energy states, charge trapping (de-trapping) phenomena, and electrochemical effects, can influence the sensed response. We then proceeded to initiate hysteresis loops by means of tip-induced spectroscopic cycling (i.e., “wake-up”), thus observing the onset of oxide degradation processes associated with this step. Finally, direct piezoelectric effects were studied using the high pressure resulting from the probe’s confinement, noticing the absence of a net time-invariant piezo-generated charge. Our results are critical in providing a general framework of interpretation for multiple nanoscale processes impacting ferroelectricity in doped-hafnia and strategies for sensing it.

## 1. Introduction

Ferroelectric (FE) doped-hafnia (HfO_2_) holds promise as a lead-free material to reignite integrated ferroelectrics, enabling low-power, high-density non-volatile memory, and integrated sensors [1]. This has yielded a growing interest from both academic and industrial communities exploring theoretical and practical aspects of FE-HfO_2_, with demonstrations of scaled ferroelectric field effect transistors (FeFET) and random access memory (FeRAM), in planar and vertical architectures [2,3,4,5,6]. More generally, hafnia is one of the most studied binary oxide systems, it is compatible with existing complementary metal-oxide-semiconductor (CMOS) process technologies and is widely used as a gate dielectric in transistors. Thus, it is available for deposition in almost every semiconductor fabrication facility. The monoclinic phase in hafnia can be reduced by incorporation of doping, capping layers, and thermal annealing, thereby increasing the presence of non-centrosymmetric orthorhombic phase that determines the appearance of a bi-stable, non-volatile electrical polarization. Ferroelectricity in ultrathin doped-HfO_2_ has been induced by the addition of various dopants such as Al, Si, Ge, Y, and N, to name but a few [7], or by using nanolaminates [1,8]. While the main deposition technique remains atomic layer deposition (ALD), epitaxial growth of doped-HfO_2_ has been recently explored using pulsed laser deposition (PLD) [9,10]. Previous studies have addressed the ferroelectric behavior in doped-HfO_2_ as a function of dopant concentration, phase composition, thermal treatment and strain, among others [7,11,12]. Interestingly, dopant-free HfO_2_ can also show ferroelectric effects which are attributed to a size induced phase transition governed by surface energy effects [13]. The majority of reports make use of macroscopic electrical characterization to determine the presence of ferroelectricity, this includes polarization-voltage (P-V) hysteresis extracted by current-voltage (I-V) characteristics in metal-insulator-metal (MIM) and metal-insulator-semiconductor (MIS) structures. In practice, most of the experiments are performed using standalone ferroelectric capacitors and require the presence of electrodes (TaN, TiN or Poly-Si are commonly used). Prepared by ALD, the FE-HfO_2_ films are polycrystalline in nature, thus containing a mixture of crystallites with different phases such as monoclinic, tetragonal, cubic and orthorhombic (m-, t-, c- and, o-phase) [1,14]. During the crystallization anneal, it has been shown that the electrodes reduce the fraction of m-phase in the oxide, likely due to the stress exerted on the film [15,16]. However, electrodes also play a major role in the unintentional incorporation of dopants, formation of interfaces and dead layers, occurrence of strain, and appearance of anti-ferroelectric phases, to name but a few. These can influence intrinsic processes in ultrathin dielectrics, such as electronic leakage, existence and generation of bulk, interface and border energy states, charge trapping (de-trapping) phenomena, and electrochemical effects such as nanoscale redox processes [17,18,19].

Information on the nanoscopic properties of FE-HfO_2_ have been revealed using scanning transmission electrons microscopy (STEM). For example, in Gd-doped HfO_2_ non-centrosymmetric o-phase was detected by STEM along different crystallographic directions [12]. Shimizu and co-authors have used annular bright-field STEM to show the change in phase for substituted epitaxial Y_x_O_1.5-(1-x)_HfO_2_ indicating the growth of o-phase film with a value of x = 0.07 [9]. Grimley et al. used TEM combined with impedance spectroscopy to study the impact of high quality interfaces and electrodes on the P_r_ for Gd-doped HfO_2_ [20]. While these works highlight the correlation between FE effects and crystalline phase, it remains very challenging to understand the intrinsic ferroelectric effects in the FE-HfO_2_ and to obtain local information on the material level not averaged by the presence of electrodes and interfaces. Additional access to nanoscopic details is required to interpret (a), the appearance of intrinsic ferroelectric properties, (b) the local mechanisms of domain formation and polarization reversal, and (c) the connection between atomistic scale processes and quantitative device operations. The understanding of these aspects can provide a correct physical picture of domain growth dynamics, fatigue and wake-up effects, a full understanding of which would enable a reduction in device variability [21,22].

**Figure 1:**
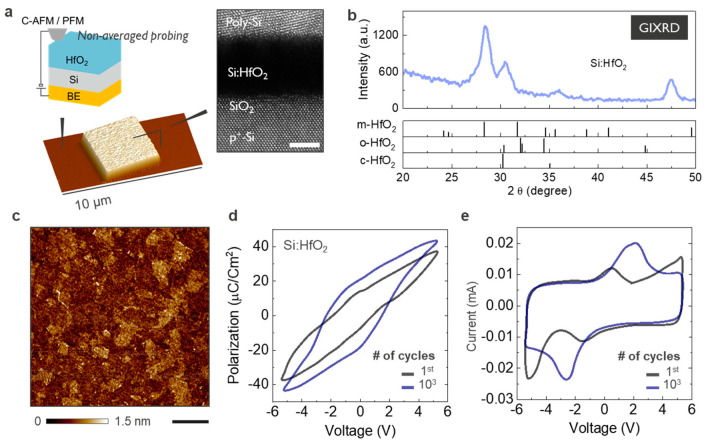
Ferroelectric phase characterization. (**a**) Schematic of the tip-sample system. The AFM image shows a fully fabricated 5 × 5 µm^2^ FE capacitor (z-scale 100 nm). The inset shows a HRTEM cross-section of the FE capacitor, 8 nm FE film is sandwiched between the Si and Poly-Si top electrode (scale bar 5 nm). (**b**) Grazing incidence X-ray diffraction patterns indicate the occurrence of the polycrystalline phases in the FE-HfO_2_ thin film after annealing. (**c**) AFM image of the area under study, i.e., away from the top electrode (scale bar 1 µm). (**d**) Polarization-voltage (P-V) and (**e**) corresponding current-voltage (I-V) characteristics for the 8-nm thick FE-HfO_2_ MIS capacitor.

Here, in the attempt to isolate intrinsic effects in FE-HfO_2_, while probing local material properties with high resolution, we investigate Si-doped HfO_2_ (FE-HfO_2_) using a wide range of scanning probe microscopy (SPM) methods. By using a nanosized probe as a movable contact, these techniques have proved themselves among the best to study FE effects, combining nanometric spatial resolution with localized sensing of surface potential, leakage current, and converse piezoelectric effects, to name but a few. In contrast with electrode-averaged characterization, SPM measurements can be performed with the probe in direct contact with the oxide surface (Figure 1a), thus creating an ultra-small capacitor with the bias applied directly to the tip-sample system (inset Figure 1a). This basic configuration is used throughout the manuscript to perform a series of different techniques using the Si-doped HfO_2_ as a technologically relevant model system. First, we study the impact of tip-induced dc polarization with readouts of piezoresponse force microscopy (PFM) performed near contact resonance. Here, we obtain a clear understating for the interference of electronic leakage with the range of dc polarization accessible by the probe. Second, band-excitation piezoresponse force microscopy (BE)-PFM and contact Kelvin probe force microscopy (cKPFM) are used to explore the possibility of ferroelectricity [23,24]. We show that the enhanced sensing capability of BE-PFM allows for probing functional details of the FE-HfO_2_ with improved resolution, while cKPFM provides evidence of the high density of bulk and interface energy states contributing to charge trapping (de-rapping) and internal screening [25]. Finally, conductive atomic force microscopy (C-AFM) and direct piezoelectric force microscopy (DPFM) are combined to sense respectively the leakage associated with charge-trapping in the oxide, and the charge induced by the direct piezoelectric effect [26,27].

Standalone FE capacitors with 8 nm FE-HfO_2_ are fabricated on p-type Si substrate (10^19^ at/cm^3^) (Figure 1a). Our material is deposited by ALD at 300 °C, the precursors employed are HfCl_4_ and SiCl_4_ as Hf and Si sources and H_2_O as oxidant. The pulse ratio of Si:Hf = 1:11 ensures a final concentration of Si dopant in range of 4 at% Si in the doped HfO_2_. The stoichiometry is confirmed by Rutherford back scattering (RBS) with elastic recoil detection (ERD), not shown. After the oxide is formed, 50 nm thick highly doped n-type Si capping layer is deposited by physical vapor deposition. The whole stack is annealed using a 1000 °C for 30 sec for the oxide crystallization. After the annealing, the Si capping layer is patterned thereby creating individual capacitors of various sizes with reactive ion etching (e.g., 5 × 5 µm^2^ imaged by AFM in the inset Figure 1a). A schematic of the sample structure is shown in Figure 1a. Cross-sectional HRTEM image of the capacitor is shown in the inset of Figure 1a, confirming the layer thickness, the sharp interfaces, and the polycrystalline nature of the film (see the Supporting Information Figure S1). As described elsewhere the presence of Si is crucial to obtain the orthorhombic phase after crystallization, we also use a capping layer (doped Poly-Si) to minimize the presence of monoclinic phase [22]. Grazing incident X-ray diffraction (GIXRD) measurements are performed with a Jordan Valley JVX7300M (Bruker Semiconductor AFM, Santa Barbara, CA, USA), monochromatic Cu Kα radiation (Ge monochromator) is used at a grazing angle of 0.5°. The spectrum is acquired after crystallization, confirming they have a high degree of crystallinity (Figure 1b). The phase ratio is typical for the imec flow and is quantified by Rietveld refinement of the grazing incidence X-ray diffraction. Furthermore, the phase composition has been seen to be strongly linked to electrode size, shape, electrode pad density, and thickness. Conventional P-V hysteresis and I-V loops are acquired on capacitors with size 100 × 100 µm^2^ and shown in Figure 1d,e. To summarize, electrically extracted values for remnant polarization (P_r_) and coercive field (E_c_) are 20 µC/cm^2^ and 2.6 MV/cm respectively. Our devices show a 10^5^ cycles endurance associated with a slight wake-up effect, where an initial increase in the remnant polarization is followed by a stable cycling behavior, as reported elsewhere [22]. Figure 1c shows the morphology of the FE-HfO_2_ in the area where the electrode has been removed. This is the area of interest in this study as we use the probe directly in contact with the oxide surface. Note that the presence of a top electrode between the tip and oxide largely suppresses the spatial variability in the output of the SPM techniques. For example, in the PFM case, when the thickness of the top electrode is non-negligible compared with the thickness of the oxide, this limits the sensing capability of local surface deformations [28]. Therefore, the generated contrast is no longer a function of the tip position and the electrode thickness determine the minimal distinguishable size of a domain [28]. Similarly, for C-AFM and DPFM both techniques lose their spatial variability if the generated charges are averaged by a metal electrode [27].

## 2. Methods

Multiple AFMs were used for this study, namely a commercial Icon PT (Bruker, Santa Barbara, CA, USA) equipped with C-AFM sensor was used for point-spectroscopy experiment and conventional PFM. A Cypher AFM system (Asylum Research, Santa Barbara, CA, USA) was used for the BE-PFM ad cKPFM experiments. The BE-PFM readout is performed by applying an ac voltage (500 mV amplitude) and exciting a band of 100 kHz around 310 kHz. The probes used are Pt-coated cantilevers with spring constant 2.8 N/m and free resonance frequency of ~75 kHz (PPP-EFM Nanosensors, Neuchâtel, Switzerland). For DPFM we used two operational amplifiers (ADA4530-1) from (Analog Devices Inc., Norwood, MA, USA) in combination with full-Pt probes (Rocky Mountains nanotechnology, Holladay, UT, USA) with spring constant 80 N/m. A commercially available PPLN sample (Bruker, Santa Barbara, CA, USA) was used for calibration of the DPFM measurements. All measurements were performed at room temperature in ambient conditions. Grazing incident X-ray diffraction (GIXRD) measurements were performed with a Jordan Valley JVX7300M (Bruker, Santa Barbara, CA, USA), monochromatic Cu Kα radiation (Ge monochromator, Bruker, Santa Barbara, CA, USA) was used at a grazing angle of 0.5°. The films were studied by grazing incidence X-ray diffraction, and the patterns were fitted using a Rietveld refinement for phase ratio. Maud software was used for this. The starting structures used in the refinement were taken from the ICSD crystallographic database in the form of .cif files for orthorhombic, monoclinic, cubic, and tetragonal pure HfO_2_, along with the .cif for the electrode material too. The phase ratio presented here is accurate within ~9%.

## 3. Tip-Induced Polarization and Multi-Domain Structure

**Figure 2:**
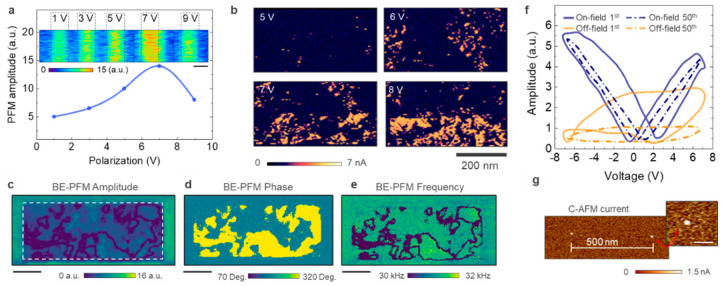
Band-excitation and C-AFM analysis results. (**a**) Non-monotonic dependence of the PFM amplitude response as a function of the dc voltage used for the poling. The inset shows regions polarized at different conditions, visually resulting in the same effect (scale bar 2 µm). (**b**) C-AFM is used to quantify the electronic leakage associated with different dc stress conditions. (**c**) Amplitude (**d**) phase and (**e**) frequency channels of BE-PFM for the 8-nm thick FE-HfO_2_ surface after dc polarization at 7 V for two scans inside the area marked by the white dashed line. The topography appears homogeneous within the investigated areas before and after dc polarization (scale bar 400 nm). (**f**), Out-of-plane BE-PFM hysteresis loop for FE-HfO_2_, comparing on-field and off-field response for the first and fiftieth cycles. (**g**) Percolative paths are observed in the location of tip-induced cycling, C-AFM is used to detect the high leakage associated with the spots at 1.5 V bias applied to the sample.

To investigate the PFM response of the bare FE-HfO_2_, the tip is first used to apply a local dc voltage to the surface. This step is required to induce the orientation of eventual ferroelectric domains and is followed by a readout step. Figure 2a shows the PFM response obtained as function of different dc polarization conditions. Multiple adjacent regions are polarized (inset Figure 2) and the average value of PFM readouts is plotted as a function of the dc bias. The observed trend is clearly non-monotonic with the PFM response showing a maximum around 6–8 V before decreasing with higher values. The origin of the observed decay can be related to the electronic leakage associated with the increasing dc stress in the FE-HfO_2_. The same dc polarization conditions were investigated on the HfO_2_ surface with C-AFM (Figure 2b). We observed a few localized conductive paths, even with voltages as low as 4–5 V applied. These paths are the results of electronic transport between the tip and the biased Si substrate, in correspondence of bulk and interface defects states in the dielectric. The tip geometry is responsible for a strong confinement of the electric field that favors the injection of electrons in these locations. The density of the leakage paths increases rapidly with the bias, as expected from dielectric degradation theory, leading to a uniform and severe electronic leakage on the surface in the range 8–10 V [29]. Therefore, under these conditions, the capability to efficiently apply a strong electric field in the FE-HfO_2_ for the domain orientation is limited by the appearance of electronic leakage in thin oxides. This establishes an optimal range for the tip-induced polarization, which in this case is assumed to be between 6 and 8 V. Noteworthy, when using the optimal conditions, the generated contrast in PFM readout appears as uniform and sharp in correspondence of the polarized area. If we consider previous studies indicating the orthorhombic phase of HfO_2_ as the major contributor for the formation of a built-in electric dipole, this is an unusual observation (as we have a nominal content of o-phase ca. 45% from GIXRD) [30,31]. Instead, independently from the percentage of orthorhombic phase in the sample, the PFM response results in a complete and uniform readout (as in see the Supporting Information Figure S2). In other words, even though only the volume fraction of material containing orthorhombic phase should provide contrast, the entire pre-polarized area presents a net visible contrast. In agreement with previous reports on the limitations of PFM for the analysis of thin films, this indicates that our attempts to use PFM in ultrathin doped-HfO_2_ are affected by severe electrostatic parasitic, with the response likely dominated by cantilever motion arising from long range electrostatics [32,33,34]. It must be noted that the applied polarization is a static dc stress applied by the probe, thus representing a different stress condition compared to what is commonly associated with the increase in volume fraction of orthorhombic phase by field-induced conversion or domain de-pinning [35].

To enhance sensing capabilities, we select BE-PFM. In contrast with traditional PFM, here ac excitation is used over a selected frequency band that is close to the contact resonance of the tip-sample system [23]. The dynamic tip-sample interaction can be approximated by a simple harmonic oscillator (SHO) model whose parameters such as resonant frequency, amplitude at resonance, phase and quality factor describe the energy dissipated due to tip-surface interactions. In case of ferroelectric materials, BE-PFM allows measurement of the amplitude and phase response of the cantilever in a frequency band encompassing the resonant peak. This approach allows extraction of the piezoresponse amplitude and phase at the resonance frequency where the signal to noise ratio is highest, even if the resonance peak shifts within the sample area. Moreover, the increased strength of the acquired piezoresponse signal, enables lower voltage values of the ac readout while achieving good signal to noise. Finally, by normalizing the piezoresponse using the SHO fitting model, BE-PFM minimizes the crosstalk between PFM and topographic features. For more details on the BE-PFM method the reader is directed to various dedicated works [23,36,37].

Figure 2c–e show the result after applying a dc voltage (7 V) for two scans. The BE-PFM readout is performed by applying an ac voltage (500 mV amplitude) and exciting a band of 100 kHz around 310 kHz to a conductive tip (PPP-EFM Nanosensors, 2.8 N/m, 75 kHz) and recording the deformation of the surface due to the converse piezoelectric effect. In contrast with the results from conventional single-frequency PFM (Supporting Information Figure S2), Figure 2c,d shows amplitude and phase contrast from isolated regions of the polarized area (i.e., dashed white line). Isolated domains are now visible with distinctive domain walls (DWs) separating them from regions that despite the dc polarization do not orient according to the applied voltage, as visible by the phase channel. Figure 2e shows the local change of the SHO fitting for the resonance frequency in the same area. This indicates the evolution of the peak position for the contact resonance and is used here for convenience to identify distinctively the position of domain boundaries. The domain walls associated with the grain boundaries of adjacent Si-doped HfO_2_ crystals can be detected with high resolution (~10 nm) as visible in Figure 2e. It is worth noting that the results in Figure 2c–e are acquired for a FE–HfO_2_ with a 45% nominal content of orthorhombic phase (by GIXRD). As visible in Figure 2d, the contrast observed in the phase signal covers almost 50% of the polarized area, likely corresponding to the domains that contain orthorhombic phase, as opposed to the non-polar regions that can be associated with monoclinic, tetragonal or misaligned orthorhombic phase. It can be expected that even among FE-phases the variance in the grains size, remnant polarization and orientation can introduce a slightly different response at different stress conditions and prolonged cycling stages (not shown here).

The experimental observations in Figure 2 are consistent with a framework of interpretation that assumes the presence of a blend of phases and domains with different polarization directions, dielectric strength, built-in fields, and coercive fields [38,39]. Due to the high density of bulk and interface defects, energy states and localized charge trapping (de-trapping) phenomena, FE-HfO_2_ is very prone to the insurgence of electrostatic artefacts when using PFM methods. Although not completely immune to these issues, the recovered contrast at high lateral resolution in BE-PFM should be ascribed to the increased sensitivity of BE-PFM combined with the use of relatively lower ac readout leading to a reduced interaction of the probe with the oxide traps. It should be also mentioned that minimal local current changes on the FE-HfO_2_ surface (Figure 2b) can contribute to the BE-PFM contrast, thus explaining the slight disagreement between GIXRD and BE-PFM. Finally, in FE-capacitor switching, wake-up and fatigue effects are consistently encountered during field cycling. These effects have been often explained as bulk and interfacial structural phase changes, with the formation of orthorhombic phase induced by cycling [20]. Clearly, in our case, the tip-induced dc polarization cannot be considered to mimic a wake-up process. Therefore, the relative fraction of active material can likely increase by a prolonged dc stress or pulsing conditions. In the attempt of detecting a tip-induced wake-up effect, we combined BE-PFM switching spectroscopy with C-AFM. In a spectroscopy experiment, a series of voltage bias pulses are applied to the tip-sample system with the probe in the same position. The BE-PFM response is measured during the application of a pulse (on-field) and in between consecutive pulses (off-field), afterward C-AFM is used to scan the area containing the spectroscopy spots. Figure 2f shows the differences in out-of-plane BE-PFM hysteresis loops for the first and fiftieth pulse. Interestingly, after a few cycles a clear closure of the hysteresis loops is observed for both on- and off-field response. We repeated the switching spectroscopy cycling in multiple locations separated by 500 nm and used C-AFM to inspect these spots (BE-PFM phase traces available in Supporting Information Figure S3). Figure 2g shows the C-AFM results, here in correspondence of two spectroscopy spots the formation of two highly conductive percolative paths are observed. The spots (Figure 2g) with size in range of tens of nm, can sustain enough current to nullify any attempt of probing field-induced polarization reversal, leading to the BE–PFM hysteresis collapse. Once more this observation must be interpreted considering the local oxide degradation that is induced by the high electric field inside the oxide on continued voltage stress. Given the thickness of the FE-HfO_2_ the value of the coercive field is dangerously close to the bias required for the oxide breakdown. When breakdown conditions are met, different mechanisms contribute to the physical modification of the oxide structure, i.e., temperature-activated oxygen vacancy migration, atomic diffusion by thermal gradient migration, to name a few [40,41]. Consequently, while the use of a conductive probe in direct contact with the oxide represents a valid solution to sense the pristine distribution of ferroelectric domains, the strong electric field confinement must be considered when attempting point-contact tip-induced switching experiments. It should be mentioned that recent reports suggest that the ferroelectricity in FE-HfO_2_ could be induced by the oxygen vacancies, whose segregation at interfaces and grain boundaries can play a major role for polarization hysteresis [42]. In this scenario, the probe being a non-blocking contact, a strong interaction of oxygen vacancies with the environment should be expected for the presence of water and oxygen radicals. Therefore, with the objective of tracking dynamic domain evolution, or operando analysis over hundreds of cycles, a passive BE-PFM analysis in the presence of a thin top electrode is likely to be more recommended, as recently reported by Chouprik and co-workers [35].

## 4. Contact-KPFM, Fixed Charges and Trapping/De-Trapping Phenomena

**Figure 3:**
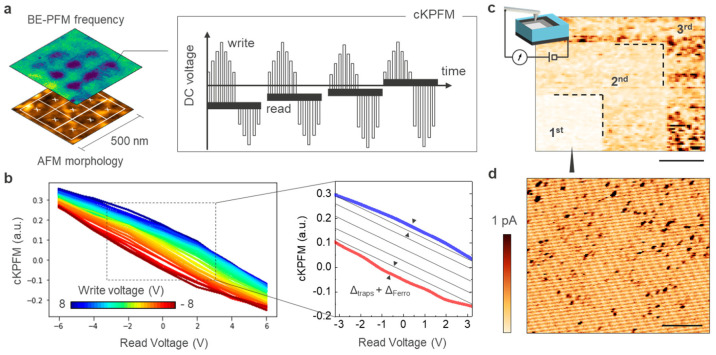
Contact Kelvin probe force microscopy analysis. (**a**) Schematic of the cKPFM spectroscopic array of points with associated pulsing scheme. (**b**) cKPFM results on the 8-nm thick FE-HfO_2_ surface, the inset is a magnification showing the −8 and +8 writing conditions with linear trendlines. (**c**) C-AFM current map acquired after three scans at increasingly large scan area with constant bias 20 mV (scale bar 1.3 µm). (**d**) C-AFM current map of the first region as measured on the first scan (scale bar 150 nm).

Although BE-PFM correctly captures the contrast between the different regions of the polycrystalline oxide, the contrast formation mechanisms of the technique does not allow electrostatic artefacts in the results to be excluded, as shown by Balke and co-authors in the case of electrochemical effects and surface charging [43]. The same authors proposed cKPFM as an alternative method to distinguish between electrostatic effects, charge injection phenomena, and ferroelectric behavior. The cKPFM mode is a spectroscopic method, that uses the sequential combination of ‘writing’ and ‘reading’ voltage pulses applied to the tip-sample system [24]. For each write voltages, a series of read signals are recorded using the aforementioned BE-PFM approach. The V_write_ pulses define the range of voltages in which the electrostatic and ferroelectric effects will be differentiated. The V_read_ are pulses with relatively smaller amplitude and are used to sense the change in contact potential difference (CPD) in the tip-sample junction that are induced by the V_write_. The technique alternates a dc voltage sweep at progressively higher amplitudes with the measurement of the change of CPD by means of small amplitude read steps (inset Figure 3a). To enable this, a probing ac signal is constantly applied on top of the ‘writing’ and ‘reading’ pulses and the cantilever response is detected using the BE–PFM scheme. As described earlier, the ac voltage frequency consists of a band of frequencies around the value of contact resonance. In a cKPFM experiment the V_write_ and V_read_ are sequentially alternated and swept in the range of choice. Therefore, one can sense the progressive change in the surface potential that is induced after each V_write_ is ramped. To some extent, the technique can be considered as the combination of (1) Kelvin probe force microscopy (KPFM) for the underlying analysis of surface potential, and (2) on-field voltage spectroscopy using BE-PFM. All performed with the probe in contact with the surface, thus the name.

Due to charge trapping processes, the CPD changes hysterically as function of the dc voltage stress. However, the shape of the hysteresis can be used to define the relative contribution of electrostatic versus ferroelectric effects [24]. A pure electrostatic signal will be linearly dependent on the applied dc voltage, thus parallel lines originating when plotting the cKPFM responses as a function of the read-out voltage (inset Figure 3b). The slope of the curves will have a correlation with the dielectric properties of the oxide (i.e., the capacitance gradient of the tip-sample system), the contact stiffness and the cantilever’s resonance properties [23]. Here, the presence of defects states in the oxide impacts the parallel lines (i.e., separation and slope), this can be related to the tendency of the oxide to trap and de-trap charges. These processes can be originated by various mechanisms such as injection in existing traps, defects generations or ionic electromigration to name a few [43]. The final result remains that for each write conditions the linear dependency of the cKPFM signal is offset by the amount of change in the CPD (inset Figure 3b). On the contrary, in the presence of remnant polarization in the bulk of the oxide, as for genuine ferroelectrics, the cKPFM curves appear as strongly non-linear with a clear remnant offset in 0 V due to the existence of a permanent polarization in the oxide. In addition, the cKPFM curves originating from different write voltages tend to form two bands with distinctive offset voltages associated with the two polarization states. The clear distinction between the two types of curves has been used as an indicator of ferroelectric materials in contrast to purely electrostatic effects. Figure 3b shows the cKPFM results obtained on the FE-HfO_2_ using a write voltage between −8 and +8 V and read pulses from −6 to +6 V. The number of points for the spectroscopic acquisition is a grid of 3 × 3 points in a 500 nm area, i.e., each point is separated by ca. 150 nm. The probing ac voltage is 2 V_pp_. Two possible situations can be detected, (i) cKPFM curves are simply linear suggesting a purely electrostatic behavior (Supporting Information Figure S4); and (ii) formation of nonlinear curves presenting a small offset value in 0V, but without the formation of clear bands (Figure 3b). If the results obtained for the first category, can be explained by assuming the response of paraelectric phases such as monoclinic, tetragonal or misaligned orthorhombic. The other locations deviate from the conventional cKPFM results obtained for ferroelectrics (inset Figure 3b), by the appearance of a clear deviation from a linear trend. While the existence of a remnant offset value in 0 that deviates from a simple linear trendline, indicates that the CPD is constituted also by an additional component (Δ_traps_ + Δ_ferro_ in the Figure 3b inset). The absence of bands can be explained by contribution of charge trapping processes to the CPD changes rather than ferroelectric polarization switching. In our interpretation, the origin of the results in Figure 3b can be ascribed to the combination of (i) relatively weak remnant polarization in FE-HfO_2_, (ii) high occurrence of trapping/de-trapping events during the cKPFM sweep. It is worth mentioning that a weak remnant polarization is not only an intrinsic material property, but in this configuration is caused also by the absence of the top electrodes introducing uniaxial strain in the film.

The high density of trapping/de-trapping defects, homogenously distributed in our film can be inferred in Figure 3c, where we record the three consecutive C-AFM scans of increasingly large dimension. Here, a minimum voltage (i.e., 50 mV) is applied between tip and sample, this aims to minimize any possible field-induced effects or electrostatic interaction. A clear (non-volatile) reduction in the electronic leakage is observed when scanning the same area multiple times. When the tip scans the surface, an extremely small current is exchanged between tip and electrically active defects. It can be easily shown that on scanning progressively larger regions the current is detected only in the area probed for the first time (Figure 3c). Already on the second scan this effect fades, as the charged defects are no longer available for electrons transport. As visible in Figure 3d, the electrically active defects are homogenously distrusted on the surface and localized in highly conductive spots of dimension ca. 10 nm. Here, the advantage compared to fully-fabricated cells is the non-averaged probing of electronic leakage virtually occurring underneath the top-electrode. For comparison, Figure 3d shows the first passage on a small scan area reported in Figure 3c, indicating (1) a high density of electrically active energy states within the bandgap for the pristine layer, and (2) a strong suppression of the electronic leakage between consecutive scans. Previous studies focusing on HfO_2_-based gate-stacks, have shown that variation in the local capacitance, oxide thickness and fixed charges can introduce trapping/de-trapping phenomena also for amorphous films [24]. The presence of these electrically active defects should not be underestimated in the attempt to probe ferroelectricity in thin oxide layers, as their state can affect directly the main parameters of ferroelectric switching by a local shift in the coercive field, relaxation processes, and dielectric degradation, but also introduce artefacts in the readouts of various PFM techniques. In addition, it is important to mention that the results shown in Figure 3c,d are acquired with a relatively small dc bias (i.e., 50 mV), while the coercive voltage measured by P-V loops is close to the breakdown voltage (i.e., a few volts at least). Therefore, when the tip is used for the local dc polarization in such thin layers, strong degradation of the dielectric properties should be expected. The data acquired in Figure 3c,d is obtained by a custom low-noise amplifier sensitive to very low level of leakage current. This is a custom-developed transimpedance amplifier, details available elsewhere [27], with a low noise level estimated from current spectral density as 1.28 fAHz and capable of probing the small charges exchange that occur between tip and sample when the tip scans a pristine surface for the first time.

## 5. Sensing the Converse Piezoelectric Effect Using DPFM

**Figure 4:**
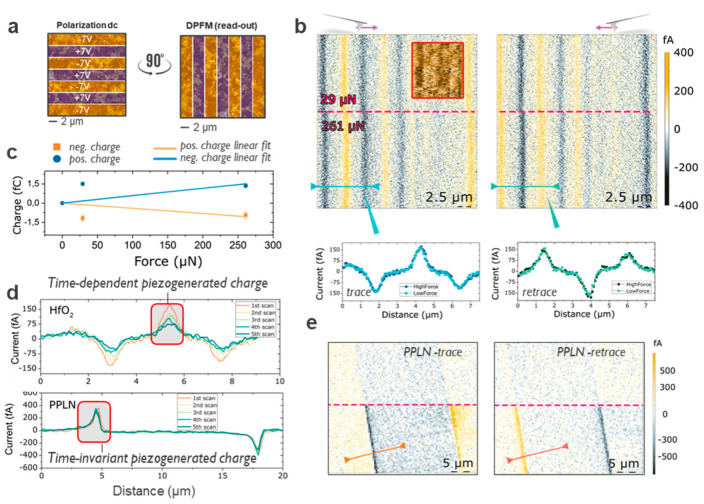
Direct piezoelectric force microscopy analysis. (**a**) Schematic of the tip-induced polarization required to detect contrast in DPFM on the FE-HfO_2_. (**b**) DPFM results on FE-HfO_2_, the load force is changed in the middle of the scan. (**c**), DPFM integrated charges as a function of the probe load force. (**d**), Comparison between HfO_2_ and PPLN, a clear difference in time-stability is visible for the piezo-generated charge, note in Figure S5e we show for PPLN the trend between pressure and generated charges which is absent in HfO_2_. (**e**) For reference we show DPFM results for PPLN, here the load force is changed in the middle of the scan.

A possible alternative to minimize the impact of undesired electrostatic and dielectric degradation effects is represented by the direct piezoelectric force microscopy method. In fact, ferroelectrics represent a sub-set of piezoelectric materials, thus they can generate electrical charge under the effect of a mechanical strain applied. This property is exploited by DPFM to sense the amount of charge generated by the application of a mechanical load. The technique probes the linear relation between mechanical deformation and piezo-generated electrical charge in piezoelectric materials [27]. Importantly, by using mechanical means, this method minimizes the possible electrostatic interaction between tip and sample, thus reducing undesired effects connected with electrically active defects. Clearly, the range of load force applicable by the probe must be carefully selected, to remain below the plastic deformation limit of the material under study, i.e., avoiding any irreversible modification of the surface. Using a relatively high load force (ca. µN) on a confined contact area (ca. tens of nm^2^), DPFM induces a small volume change and senses the generated charges with a low noise transimpedance amplifier. Once more, low current range sensitivity is required (0.1 fA-10 pA). The use of full metal probes is indicated to avoid the loss of conductivity in coated tips used under high pressure, here we use full platinum 25PtIr300B (Rocky Mountains nanotechnology, Holladay, UT, USA). Previously, DPFM has been used to extract piezo-generated charges in conventional piezoelectric samples such as lead zirconate titanite (PZT), periodically poled lithium niobate (PPLN), and bismuth ferrite thin films [27,44]. These materials present natural domain structures that are easy to detect and allow the DPFM to sense directly the linear dependence between the piezo-generated charge and tip-induced load forces, showing good agreement with previously reported values for the extracted values of pC N^−1^. For the sake of clarity, in Supporting Materials Figure S5 we report on the comparison between PPLN and FE-HfO_2_ using the same probe and similar stress conditions. Importantly, DPFM does not provide any visible results in the range of load force between 1 and 100 µN on the pristine surface of the FE-HfO_2_. Moreover, the absence of natural domain structures (compared to PZT or PPLN) does not help in correlating morphological and electrical features. Thus, we performed a tip-induced dc polarization to define artificial zones (rectangles) of opposite ferroelectric polarization regions as in Figure 4a (V_pol_ = ± 7 V, scan angle 90°). The results of the DPFM are presented in Figure 4b, in which we perform a readout at 0° scan angle (trace and retrace are shown). As highlighted by a red dashed line, the force is drastically changed at approximately the middle of the image (Figure 4b). By integrating the current profiles, we can obtain the charges built up by the direct piezoelectric effect, for each of the different peaks, see inset Figure 4b. Expecting a linear dependence of the charge with applied force, we averaged the charges obtained from each of the peaks, and plotted as a function of applied force, see Figure 4c. A linear fitting is used to try to correlate the charge with applied load. However, this linear fitting cannot be realized (R^2^ = 0.34) concluding that the generated charges collected are not dependent upon applied force.

As a comparison, we performed a similar set of experiments with a known ferroelectric material such as PPLN as Figure 4d,e. It is clearly observed that the charge generated signal changed dramatically with the force increase (Figure 4e). In addition, from the integration of the profile, the generated charge is obtained and plotted as a function of the applied force (Supporting Information Figure S5e). In this case, a linear equation perfectly fits our results (R^2^ = 0.99) confirming our expectations with an appreciable increase or dependence of the force applied. Therefore, the absence of a linear dependency between recorded charges upon applied force suggests that the nature of the contrast, does not originate through a piezoelectric effect. This is supported by the results in Figure 4d where we study the time-evolution of the DPFM signal for FE-HfO_2_ and PPLN. Trace and retrace profiles from each of the scanned frames are extracted and averaged. The profiles in Figure 4d show charges as obtained by integration of the current profiles. While the PPLN signal is constant as function of repeated scans, the generated charges in FE–HfO_2_ clearly decay. In five consecutive scans, the charge recorded diminished from −1.2 to −0.3 and from 1.5 to 0.8 fC, for both negative and positive integrated peaks. This decay cannot be explained with a ferroelectric polarization reversal mediated by flexoelectric effect which should occur abruptly upon a given force value threshold [45]. The lack of time-invariancy for the DPFM response in FE–HfO_2_ strongly suggests a non-piezoelectric nature for the observed contrast that could originate from the modification of the surface potential, induced during the phase of dc polarization, which tends to discharge on consecutive DPFM scans.

## 6. Conclusions

In conclusion, several state-of-the-art techniques have been combined to identify the challenges associated with the direct probing of intrinsic ferroelectric and piezoelectric effects in thin Si-doped HfO_2_. As shown here, by studying the FE-HfO_2_ surface without top-electrodes but using a SPM probe as a movable contact, weakly detectable evidences of intrinsic ferroelectricity can be sensed at the nm scale. In detail, we observed a good correlation between the content of orthorhombic phase in the material as measured by X-ray diffraction, and the BE-PFM analysis. C-AFM has been used for the nanoscopic visualization of the electronic leakage and charge injection, defining the optimal operation condition to avoid an excessive oxide degradation. For each method we discussed the importance of the analysis conditions, with emphasis on possible undesired coupling between mechanisms of dielectric gradation, ferroelectric and piezoelectric effects in thin oxides. As a result, while the probing of crystalline domains has been possible using BE–PFM, the role of the electrodes and interfacial layers must be considered crucial for stabilizing and preserving the formation of the ferroelectric orthorhombic phase that generates a detectable built-in electric dipole. It should be mentioned that the values required to avoid surface modification or unrecoverable oxide breakdown, can often be smaller than the coercive field, thus leading to a less pronounced hysteretic behavior. Moreover, we showed how abundant charge trapping and de-trapping events occur also at moderate bias, with impact on the readouts of the other techniques. In the case of cKPFM, the sampling volume confinement imposed by the spectroscopic sensing can play a role. In fact, even considering the probe as perfectly positioned on a well oriented orthorhombic crystal, the contribution of trap states might overwhelm the role of the ferroelectric dipole switching, thus limiting the insurgence of nonlinear cKPFM spectra with band formation. In addition, recent studies suggest that the ferroelectric contribution relies on the distribution and concentration of oxygen vacancies in the oxide [42]. Therefore, a relatively open environment such as the probe in direct contact with the oxide, would allow the oxygen vacancies to interact with other defects, absorb humidity near the sample surface, or to migrate and redistribute inside the bulk. Finally, the absence of a net time-invariant piezo-generated charge in DPFM and the lack of a linear dependency between the generated charge and the force applied, implies that the intrinsic converse piezoelectric effect obtainable must be either below the detection limit, or strongly reduced possibly due to a relaxation process that occurs to the orthorhombic crystals in absence of the top electrode. The direction of our future works will concentrate on (a) exploration of emerging methods less sensitive to electrostatic coupling, i.e., using microwaves or interferometry, (b) the use of operando analysis methods in the presence of thin top electrodes, to limit the insurgence of undesired surface energy effects, maintaining actively in place interface layers, and the mechanical strain of a full stack configuration. Due to the technological relevance for microelectronics, ferroelectric properties of doped-HfO_2_ layers have been extensively studied in recent years. However, with our results we raise awareness regarding the complexity of data interpretation, and the challenges of sensing ferroelectric properties in a predominantly non-polar surrounding.

## Supplementary Materials

This material is available free of charge via the Internet at https://www.mdpi.com/2079-4991/10/8/1576/s1. In the supporting information we report on the use of high-resolution transmission electron microscopy to study the quality of interfaces and crystallinity of the Si-doped HfO_2_. We report on a potential electrostatic artefact affecting the study of ferroelectricity using PFM and we compare the results with cKPFM for non-ferroelectric oxides. Finally, we provide details on the use of DPFM and compare the results obtained by the technique for e PPLN ferroelectric layer to identify the major difference that we show in the main text for Si:HfO_2_. Figure S1: Si-doped HfO2 measured by high resolution transmission electron microscopy (HRTEM). Figure S2: Conventional non-resonant PFM images are shown in figure, Figure S3: BE-PFM point-spectroscopy study, Figure S4: The case of cKPFM for a pure dielectric layer (undoped-HfO_2_), Figure S5: DPFM trace (a) and retrace (b) for PPLN test structure probes with two distinctive load forces. (c) PPLN extracted profiles at the domain walls as a function of the load forces, clearly indicating a pressure dependence between charge generated and force. (d) PPLN extracted profiles at the domain walls (retrace). (e) The integration of the line profiles is sued to generate a plot that relates charge as a function of applied force that shows a linear dependence for PPLN.
