# Supplementary material for: Ferroelectricity in Si-Doped Hafnia: Probing Challenges in Absence of Screening Charges
Source: Nanomaterials (Basel). 2020 Aug 11;10(8):1576. doi: 10.3390/nano10081576 (PMC7466465; doi:10.3390/nano10081576)
Supplement: Supplementary file 1 [file nanomaterials-10-01576-s001.pdf]

Supplementary Materials

Ferroelectricity in Si-Doped Hafnia: Probing Challenges in Absence of Screening Charges

Umberto Celano [1,*], Andres Gomez [2], Paola Piedimonte [1,†], Sabine Neumayer [3], Liam Collins [3], Mihaela Popovici [1], Karine Florent [1,‡], Sean R. C. McMitchell [1], Paola Favia [1], Chris Drijbooms [1], Hugo Bender [1], Kristof Paredis [1], Luca Di Piazza [1], Stephen Jesse [3], Jan Van Houdt [1,4] and Paul van der Heide [1]

[1] Imec, Kapeldreef 75, B-3001 Heverlee (Leuven), Belgium; paolapiedimonte@gmail.com (P.P.); Mihaela.Ioana.Popovici@imec.be (M.P.); kaflorent@micron.com (K.F.); Sean.McMitchell@imec.be (S.R.C.M.); paola.favia@imec.be (P.F.); Christel.Drijbooms@imec.be (C.D.); hugo.bender@imec.be (H.B.); kristof.paredis@imec.be (K.P.); Luca.DiPiazza@imec.be (L.D.P.); Jan.VanHoudt@imec.be (J.V.H); Paul.vanderHeide@imec.be (P.v.d.H.)

[2] Institut de Ciència de Materials de Barcelona (ICMAB-CSIC), Campus UAB, Bellaterra, 08193 Catalonia, Spain; agomez@icmab.es

[3] Center for Nanophase Materials Sciences, Oak Ridge National Laboratory, 1 Bethel Valley Rd., Oak Ridge, TN 37830, USA; neumayersm@ornl.gov (S.N.); collinslf@ornl.gov (L.C.); sjesse@ornl.gov (S.J.)

[4] Department of Electrical Engineering (ESAT), KU Leuven, Kasteelpark Arenberg 10, 3001 Leuven, Belgium.

* Correspondence: umberto.celano@imec.be

† Currently with Department of Electronics Information and Bioengineering (DEIB), Politecnico di Milano, Piazza Leonardo da Vinci, 32, 20133, Milan, Italy

‡ Currently with Micron Technology Inc., 8000 S Federal Way, Boise ID 83707, USA

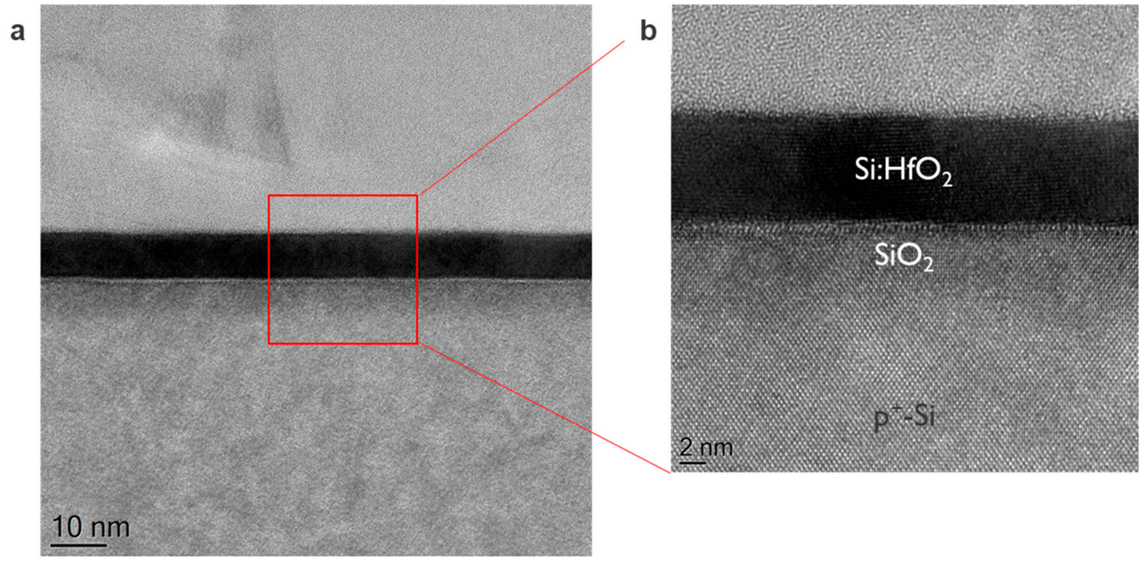

Figure S1 Si-doped HfO_2 measured by high resolution transmission electron microscopy (HRTEM), the presence of sharp interfaces (**a**) and the polycrystalline nature of the oxide layer (**b**) are both visible.

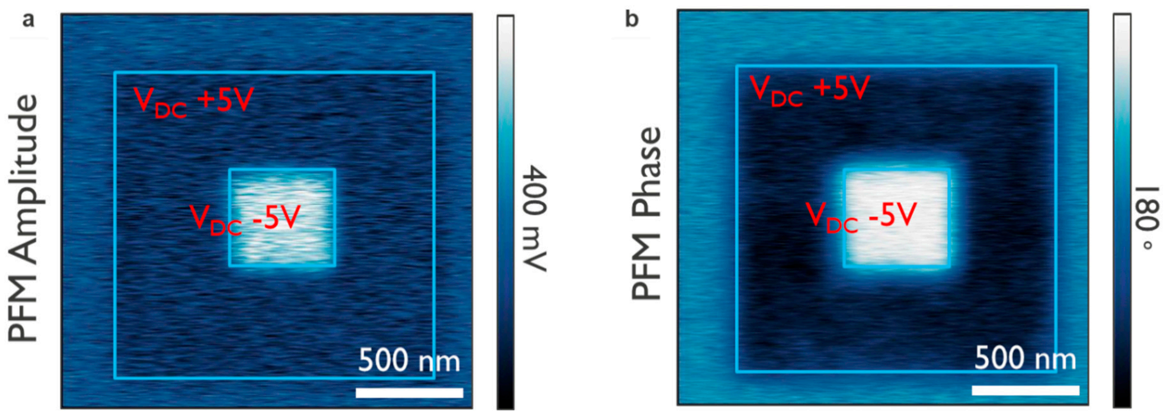

Figure S2 Conventional non-resonant PFM images are shown in figure, (**a**) amplitude and (**b**) phase contrast. The probed area has been previously polarized using two scans with a dc bias applied between tip and sample as indicated in highlighted regions. It is noteworthy that independently from the percentage of orthorhombic phase contained in the sample, the PFM response results always as a complete and uniform readout clearly suggesting a undesired surface charging-induced PFM contrast.

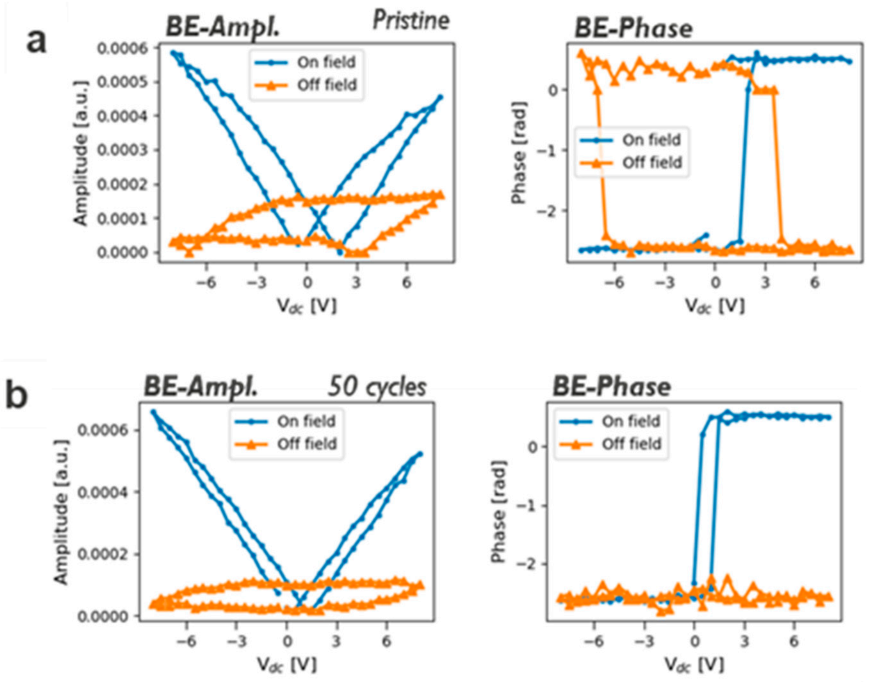

Figure S3 BE-PFM point-spectroscopy study, comparison between pristine (**a**) and cycled (**b**) Si:HfO_2. Amplitude and phase readout are presented for the same location that is stressed with multiple dc I-V sweeps with consequent collapse of the BE-PFM response.

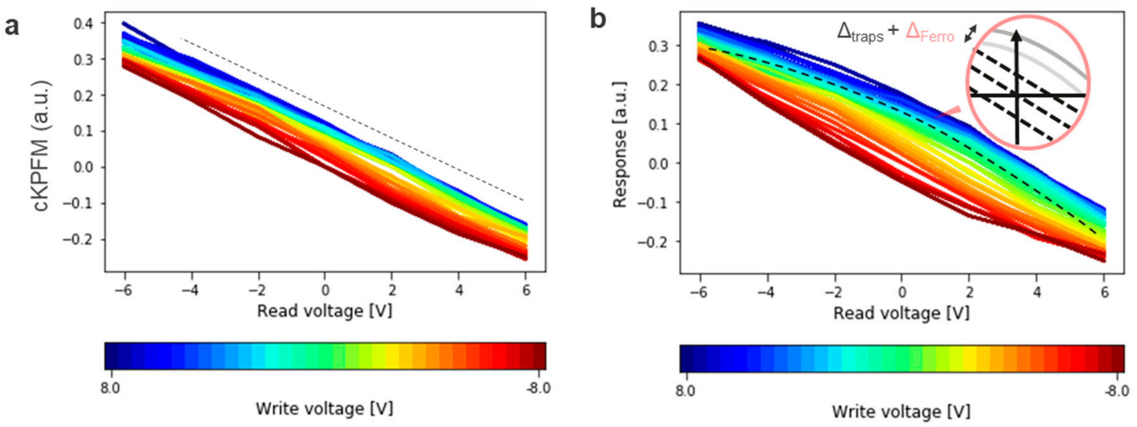

Figure S4 The case of cKPFM for a pure dielectric layer (undoped-HfO_2) is presented in (a) as a comparison for the results obtained in the Si-doped HfO_2. The two layers have the same thickness (8 nm) and the appearance of a linear dependence in the cKPFM readout suggest a purely electrostatic behavior for the undoped layer (as expected) with clear differences for the case of doped layers.

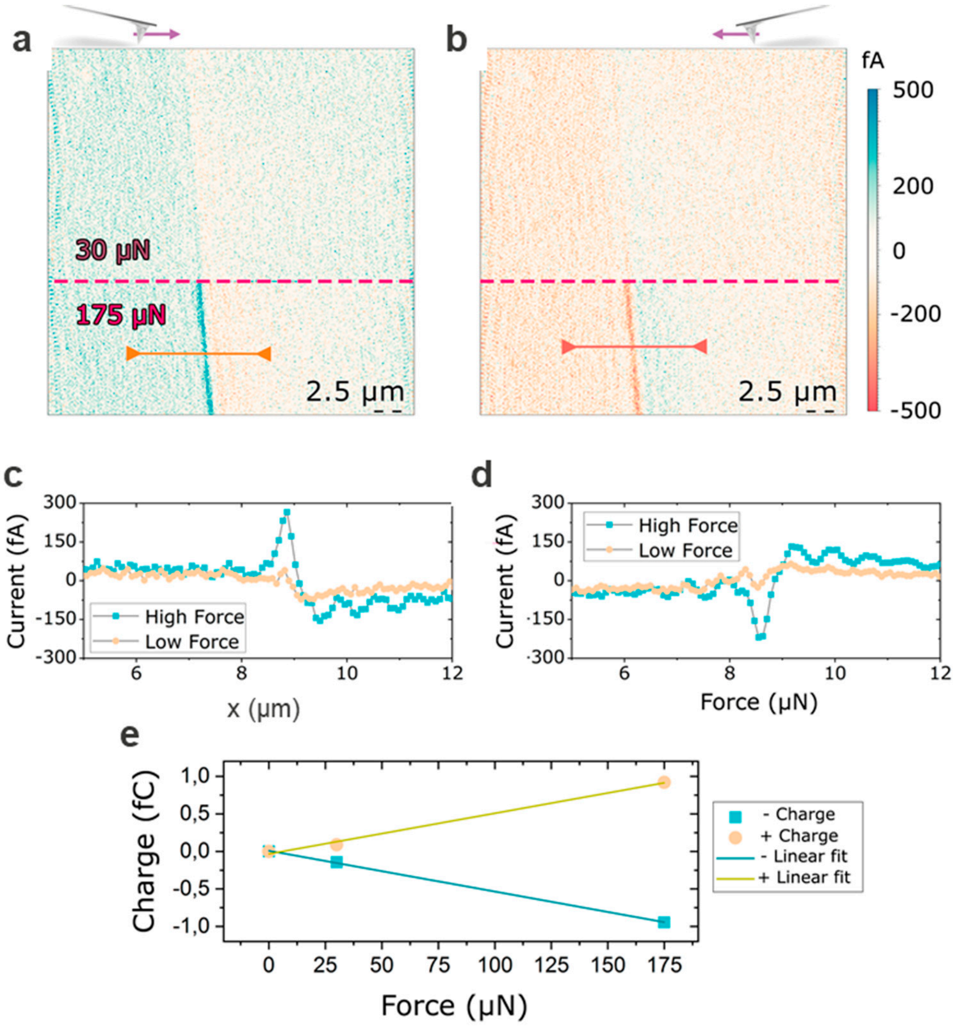

Figure S5 DPFM trace (**a**) and retrace (**b**) for PPLN test structure probes with two distinctive load forces. (**c**) PPLN extracted profiles at the domain walls as a function of the load forces, clearly indicating a pressure dependence between charge generated and force. (**d**) PPLN extracted profiles at the domain walls (retrace). (**e**) The integration of the line profiles is sued to generate a plot that relates charge as a function of applied force that shows a linear dependence for PPLN.
